# The Pathophysiological Link Between Reelin and Autism: Overview and New Insights

**DOI:** 10.3389/fgene.2022.869002

**Published:** 2022-03-29

**Authors:** Marcello Scala, Eleonora A. Grasso, Giuseppe Di Cara, Antonella Riva, Pasquale Striano, Alberto Verrotti

**Affiliations:** ^1^ Department of Neurosciences, Rehabilitation, Ophthalmology, Genetics, Maternal, and Child Health (DINOGMI), University of Genoa, Genoa, Italy; ^2^ Pediatric Neurology and Muscular Diseases Unit, IRCCS Istituto Giannina Gaslini, University of Genoa, Genoa, Italy; ^3^ Department of Paediatrics, University of Chieti, Chieti, Italy; ^4^ Department of Medicine and Surgery, Pediatric Clinic, University of Perugia, Perugia, Italy

**Keywords:** RELN, reelin, autism, ASD, brain development, GGC repeats, single nucleotide polymorphisms

## Abstract

Reelin is a secreted extracellular matrix protein playing pivotal roles in neuronal migration and cortical stratification during embryonal brain development. In the adult brain, its activity is crucial for synaptic plasticity, memory processing, and cognition. Genetic alterations in RELN have been variably reported as possible contributors to the pathogenesis of autism spectrum disorders (ASD). In particular, GCCs repeats in the 5′UTR, and single nucleotide polymorphysms (SNPs) in RELN have been suggested to affect brain development and predispose to autism. We reviewed pertinent literature on RELN expression and haplotypes transmission in children with ASD, critically analyzing available evidence in support of the pathophysiological association between Reelin deficiency and ASD.

## Introduction

Reelin is an extracellular matrix protein with serine protease activity which highly secreted in several regions of cerebral and cerebellar cortex (Fatemi, 2002). It is encoded by the RELN gene (OMIM *600514) on chromosome 7q22, whose expression is significantly regulated by complex epigenetic modifications (D’Arcangelo et al., 1995; DeSilve et al., 1997; Folsom and Fatemi, 2013). The initial protein precursor is processed and subsequently secreted as a modular glycoprotein able to bind several receptors, including very low density lipoprotein receptors (VLDLRs) and apolipoprotein E receptor 2 (ApoER2) (Fatemi, 2005). The interactions with these receptors initiate a signalling cascade that involves the recruitment of Disabled-1 (Dab1) and the activation of Src family kinases (Fatemi, 2005). This leads to the reciprocal activation of Dab1 domain, which starts itself a crucial signalling cascade for correct neuronal migration and cortical layer formation (Lammert and Howell, 2016; Armstrong et al., 2019). Of note, this cascade also results in the phosphorylation of the N-methyl-d-aspartate receptor (NMDAr) subunits NR2A and NR2B (Chen et al., 2005; Folsom and Fatemi, 2013).

In addition to a cardinal regulatory role in neuronal migration and cortical stratification during the embryonal period, Reelin is involved in synaptic plasticity, memory processing, and cognition in the adult brain (Fatemi, 2005; Armstrong et al., 2019). It is particularly abundant in the Cajal-Retzius neurons of the marginal zone in the cerebral cortex, but high levels of Reelin can be also observed in several regions of the cerebellum, including the surface of the developing cortex, the deep nuclei, and the internal granular layers, where it promotes the migration of Purkinje cells (Armstrong et al., 2019). An impaired Reelin production in the cerebral cortex may lead to cortical abnormalities (e.g., heterotopia, polymicrogyria, and lissencephaly) (Armstrong et al., 2019). In the adult brain, the protein is mainly secreted by a subpopulation of GABAergic interneurons and its distribution pattern is different in comparison to the developing brain, in line with the different functional roles (Ishii et al., 2016).

A growing interest on the pathogenic contribution of Reelin deficiency in neuropsychiatric conditions, including autism spectrum disorder (ASD), has emerged in the last two decades (Fatemi, 2005). ASD is an early-onset developmental condition of variable severity characterized by impaired communication and social interactions, and restrictive or repetitive patterns of thoughts and behaviours (Hutcheson et al., 2003). Although an underlying genetic cause is strongly suspected, the lack of evident classic mendelian inheritance mechanisms suggests a complex genetic aetiolgy (Hutcheson et al., 2003). The observation of brain volume abnormalities in patients with ASD and the reports on a possible autism susceptibility gene on chromosome 7 further stimulated the interest on Reelin as a possible disease biomarker (Folsom and Fatemi, 2013). The aim of this review was to explore the complex and controversial results achieved in the numerous studies investigating the possible pathophysiological link between Reelin and ASD.

## Methods

We analyzed the relevant studies published between 2001 and 2020 regarding the role of *RELN* in ASD. Electronic databases (MEDLINE, EMBASE, and the Clinical Trial Database) and papers from the scientific literature were systematically searched using the following terms: “reelin,” “RELN gene,” “reeler mouse,” “reeler mouse and autism,” “reelin and autism,” “reelin and autism spectrum disorder,” and “reelin and autism mouse models”. The abstracts of retrieved references were reviewed and prioritized by relevant content and quality of the reported evidence. The reference lists of the selected articles were used to search for further relevant papers. Only articles in English were reviewed.

## Results

### GGC Repeats in the 5′UTR Alleles

The first study to suggest a relationship between *RELN* alleles and ASD was conducted in 2001 by a multicenter group (Persico et al., 2001), as (Table 1). The authors studied a large cohort of 184 autistic patients and 186 controls from Italy and the United States, focusing on the families with affected individuals (172 singletons and 5 multiplex). This was the first report on a possible role of long GGC repeats (conventionally at least 11) in the 5′UTR region of *RELN* as a risk factor for autism. Family-based analysis in families with a single autistic child highlighted a more likely transmission of these large alleles to the affected offspring in comparison to healthy siblings. In the same year, another study focused on the comparison of RELN and Bcl-2 levels in homogenates of cerebellar tissue obtained from 5 subjects with autism and 8 controls, which showed a significant decrease of Reelin (43–44%) and Bcl-2 (34–51%) levels in comparison to healthy controls (Fatemi et al., 2001). These proteins are both involved in neuronal migration and cellular programmed death, and the results of this study were consistent with their dysregulation in ASD.

**TABLE 1:** Summary of the positive and negative *RELN*-ASD correlations in the published cohorts.

Reference	ASD	Controls	Country of origin (ethnicity)	Genetic abnormalities	Correlation with autism	
					Yes	No
Persico et al. (2001)	184	186	Italy, United States (NA)	5′ UTR, long GCC triplets	+	
				SNPs exon 6		
				SNPs exon 50		
Zhang et al. (2002)	235	182	China (NA)	5′ UTR, CGG/GCC repeats	+	-
Krebs et al. (2002)	218		France, Sweden, Norway, Italy, Austria, Belgium, United States (NA)	5′ UTR, CGG/GCC repeats		-
Bonora et al. (2003)	55	-	Germany (NA)	Ten missense variants identified		
	342	192	Germany (Caucasian)	Exon 10 (G1283C transversion in G370R; T1188G variant in V338G)		-
				Exon 25 (C3652A transversion in N1159K)		
				Exon 35 (G5459A transition in V1762I; C5399T variant in R1742W)		
				Exon 35 G5400A (R1742Q)		
				Exon 44 (G7044A variant in R2290H)		
				Exon 51 (A8327G transition in T2718A)		
	158	197	Germany (Caucasian)	Exon 25		-
				Exon 35		
				Exon 44		
				Exon 51		
			Germany (Caucasian)	5′ UTR		-
				Intragenic SNPs		-
Devlin et al. (2004)	42	20	NA (American)	5′ UTR and long GCC triplets		-
Li et al. (2004)		-	NA (American)	5′ UTR and long GCC triplets		-
Skaar et al. (2005)	577	97	NA (Caucasian)	GGC repeats 5′ UTR	+	
				10-repeat allele exon 6		
				exon 44		
				exon 45		
				exon 50		
				intron 59 (rs73670)		
Persico et al. (2004)	235	182	Italy (Caucasian)	RELN long repeats	+	
				APOE	+	
Ashley-koch et al. (2007)	-	-	Italy (Caucasian)	GGC repeats 5′UTR rs2073559	+	
				rs736707	+	
					+	
				APOE		-
Li et al. (2008)	213	160	China (NA)	SNP1 (rs1062831) exon 62	+	-
				SNP2 (rs736707) intron 59		-
				SNP3 (rs2229864) exon 50		-
				SNP4 (rs362746) exon 45		-
				SNP5 (rs362726) intron 31		-
				SNP6 (rs362691) exon 22		-
				SNP7 (rs2072403) exon 15		-
				SNP8 (rs607755) intron 5		
Serajee et al. (2006)	NA	NA	NA (Caucasian)	G/C SNP in exon 22 (rs362691, V997L)	+	
				C/G SNP in exon 34 (rs2229860, P1703R)	+	
				A/G polymorphism in exon 45 (rs362746, V2370V) synonymous G/A SNP in exon 48 (rs2075038, P2510P)	+	
				A/G transversion (rs607755) located in the 5v splice junction of exon 6		
				C/T SNP in intron 59		
Dutta et al. (2007)	73	80	India (NA)	CGG repeats 5′ UTR		-
				A/G transversion in exon 6 SNP		-
				T/C transversion in exon 50 SNP		-
Dutta et al. (2007)	102	101	India (NA)	(rs727531) intron 12		-
				(rs2072403) exon 15		
				(rs2072402) intron 15		
				SNP4 (rs362691) C/G transversion in exon 22		
				(rs362719) intron 41		
				(rs73670)		
				(rs73670) intron 59		
He et al. (2011)	232	283	China (NA)	rs736707		-
				rs2229864		
				rs362691		
				rs2073559		
Tian (2012)	186	181	China (NA)	g.504742G > A (exon 60)	+	-
				g.333509A > C (intron 12)		
Fu et al. (2013)	205	200	China (NA)	g.296596G > A	+	
Shen et al. (2016)	430	1,074	China (NA)	rs2528858		-
				rs1858782		
				rs7799028		
				rs634500		
				rs6943822		
Wang et al. (2018)	157	256	NA	rs2229864	+	
				rs736707/rs2229864 haplotype	+	
				rs2229864		-
				rs736707		
Kelemenova et al. (2010)	90	85	Slovakia (NA)	GGC STR polymorphism in the 5′ UTR	+	
Holt et al. (2010)	NA	NA	Europe (NA)	rs362780 (intron 39)	+	

Abbreviations: ASD, autism spectrum disorder; NA, not applicable; SNP, single nucleotide polymorphism; STR, short tandem repeat; UTR, untranslated region.

The role of CGG-repeats in the 5′UTR of *RELN* was later investigated in 126 multiple-incidence families and 68 individuals with no familiar history of ASD (Zhang et al., 2002). Fourteen *RELN* alleles with different number of CGG-repeats (3–14, with 8, and 10 repeats being the most common) were identified, in absence of evident preferential triplets expansion or unstable CGG-triplets transmission (Zhang et al., 2002). The same study tested the hypothesis, first formulated by Persico et al., that there is a higher autism risk in patients harboring larger *RELN* alleles (≥11 repeats) and that these alleles tend to be more frequently transmitted in affected children (Persico et al., 2001). Although the increased frequency of large *RELN* alleles in autistic children with negative family history could not be confirmed, an increased trend of large *RELN* alleles and genotypes in the analyzed offspring were also observed (Persico et al., 2001).

A correlation between *RELN* alleles >11 repeats and autism severity according to the ADI-R algorithm was investigated. Four different domains (qualitative impairment in reciprocal social interaction; verbal and non-verbal communication; repetitive behaviors; stereotyped patterns) were evaluated. No specific correlation in terms of severity could be observed, but patients with at least one *RELN* allele >11 repeats were found to have an earlier disease onset. On this basis, a possible role for large *RELN* alleles in some form of ASD (e.g., children without delayed speech) was hypothesized. However, in a family-based linkage/association study analyzing genotype distribution and allele frequencies of the 5′UTR polymorphism of *RELN*, the lack of a significantly increased transmission of longer GGC alleles was instead observed (Persico et al., 2001; Krebs et al., 2002). Of note, a lower frequency of the allele 12 and a new 16-repeat allele were detected in this same study (Persico et al., 2001; Krebs et al., 2002). The discrepancies between the two studies were likely explained by the heterogeneous ethnicity of the studied cohort and the clinical heterogeneity of ASD.

In a large genetic study conducted on behalf of the International Molecular Genetic Study of Autism Consortium (IMGSAC), Bonora et al. reported several *RELN* variants absent in control groups (involving exon 10, 25, 35, 44, and 51), while previously reported triplets repeat polymorphisms were not significant (Bonora et al., 2003). However, no significant linkage association in the studied families or differences in behavioral phenotypes of subjects harboring these variants emerged (Bonora et al., 2003). Devlin et al. tried to replicate previously published results analyzing a larger family-based cohort of patients with a similar allele distribution (Persico et al., 2001; Devlin et al., 2004). Interestingly, no significant linkage or association was found among *RELN* alleles, autism, and under-transmission of long alleles (Devlin et al., 2004). A similar lack of evidence was also found by an American group that studied a polymorphic trinucleotide repeat in 5′UTR of *RELN* in a cohort of 107 families (214 parents and 254 children) (Li et al., 2004). No significant differences emerged in the distribution of specific alleles or genotypes, and in the frequency of haplotypes containing larger alleles (Li et al., 2004). However, since no additional *RELN* variants were studied, it was not possible to exclude a link with other *RELN* polymorphisms.

### Single Nucleotide Polymorphisms

The previously reported statistically significant association between 5′UTR alterations in *RELN* and autism was confirmed by an interesting study by Persico et al. (2001), Skaar et al. (2005). The authors found a high expression and transmission of the allele 10 (193 transmitted *vs.* 160 not transmitted, *p* = 0.003), but also detected a significant correlation among other single nucleotide polymorphisms (SNPs) and exons in specific subsets of patients, suggesting that several distinct *RELN* haplotypes may be involved in determining ASD susceptibility (Persico et al., 2001; Skaar et al., 2005). In a second study focused on a larger population (470 subjects versus 371 of the original study), this same group investigated the linkage association between the *RELN* polymorphism rs2073559 and *APOE* (Persico et al., 2004; Ashley-Koch et al., 2007). Even if a significant association emerged, this was most likely dependent on the single locus result of rs2073559 and no correlation with GGC repeat size was detected (Persico et al., 2004; Ashley-Koch et al., 2007). A link between autism and the transmission of two SNPs [NM_173054.2: c.2989C > T; p. (Leu997Phe) (rs362691) in exon 22 and NM_173054.2: c.5108C > G; p. (Pro1703Arg) (rs2229860) in exon 34] was later reported (Serajee et al., 2006). The study involved 196 families that were tested for 34 SNPs and an A/G transversion in the 5V splice junction of intron 6 of RELN [NM_173054.2: c.578-3T > C (rs607755)] (Serajee et al., 2006). The most significant association involved the rs736707 (intron 59), but strong associations were also reported for the rs362691 (exon 22) and the rs2075038 (exon 48) (Serajee et al., 2006).

In a large Indian study, the distribution of CGG repeat polymorphisms at 5′UTR and the SNPs at exons 6 and 50 was investigated (Dutta et al., 2007). Initial data did not show a preferential transmission of any of these alleles to affected individuals (Dutta et al., 2007). Six additional SNPs were later investigated (rs727531, rs2072403, rs2072402, rs362691, rs362719, and rs736707), confirming previously published data and showing a lack of association with ASD in the Indian population (Bonora et al., 2003; Serajee et al., 2006; Dutta et al., 2007). Li et al. investigated 12 SNPs in *RELN* and *GRM8* (OMIM *601116), encoding a crucial glutamate presynaptic release protein with a possible role in ASD pathophysiology, in a cohort of 213 Chinese autistic children (Carlsson, 1998; Li et al., 2008). The authors reported a significant association between rs736707 in *RELN* and autism, while no association with other exonic SNPs was observed (Carlsson, 1998; Li et al., 2008). However, these findings were not confirmed by a further case-control study investigating four SNPs (rs736707, rs2229864, rs362691, and rs2073559) in the Han population, in which no evident association could be detected (He et al., 2011). The *RELN* polymorphisms g.333509A > C (intron 12) and g.504742G > A (exon 60) were also examined within a Han cohort of 186 patients and 181 controls (Tian, 2012). While no correlation was observed for g.333509A > C, a significant frequency difference emerged for the g.504742G > A polymorphism (Tian, 2012). Eventually, the g.296596G > A variant in *RELN* was found to exert a potential influence on autism susceptibility in a Chinese Han cohort of 205 patients (Fu et al., 2013).

Despite the controversies (Li et al., 2008; He et al., 2011), Wang et al. confirmed the previously reported correlation between *RELN* SNP rs2229864 and ASD (Chen et al., 2017; Wang et al., 2018). This was the first study to report the rs2229864-rs736707 haplotype (Chen et al., 2017; Wang et al., 2018). An intriguing object of debate remained the actual importance of the rs736707, previously highlighted by Li et al. in 2008 but excluded by He et al. in 2011 (Li et al., 2008; He et al., 2011). Although the association of this SNP with ASD risk was not confirmed (Li et al., 2008; He et al., 2011; Chen et al., 2017; Wang et al., 2018), it is interesting to note that a possible relationship between the rs736707 and the clinical features of the studied Chinese autistic children was suggested (Chen et al., 2017; Wang et al., 2018). When several candidate genes involved in the synaptogenesis were investigated in a study conducted in a Slovakian cohort, *RELN* was the only gene showing a statistically significant correlation with the ASD group (Kelemenova et al., 2010). Furthermore, the increased number of GGC repeats (12 and 13 repeats) in the 5′UTR was associated with a decreased *RELN* expression, confirming the findings of the very first studies published (Persico et al., 2001). Eventually, a multicenter European study on the possible link between candidate genes and ASD led to the identification of a positive correlation between autism and the *RELN* SNP rs362780 (intron 39) (Holt et al., 2010).

### Gene-Gene Interactions

Gene-gene interactions were explored in a Chinese study analyzing several haplotypes of genes involved in the Reelin pathway (Shen et al., 2016). No significant association with a single SNP was detected (Shen et al., 2016). However, the authors confirmed that the interaction between *RELN* and *DAB1* (OMIM *603448), encoding an intracellular adaptor which is tyrosine-phosphorylated when Reelin binds to lipoprotein receptors, may contribute to autism pathogenesis (Shen et al., 2016). Fifteen genes involved in Reelin signaling were investigated in a family-based study conducted in a further Chinese Han population (Li et al., 2013). Four SNPs in *DAB1* were found to be associated to ASD and were suggested to represent a risk factor for autism (Li et al., 2013).

## Discussion

A growing interest raised on the study of *RELN* after the cloning of the mouse *reeler* mutation (D’Arcangelo et al., 1995). In this model, there is an abnormal migration of normal appearing neurons which eventually leads to several structural defects (e.g., cerebellar hypoplasia, abnormal cell bodies orientation, deficient neuronal positioning, and inverted cortical lamination) (Falconer, 1951; Goffinet, 1979; D’Arcangelo et al., 1995; Fatemi et al., 2001; Magdaleno et al., 2002; Ohshima et al., 2002). The phenotype associated with biallelic variants in the mouse homolog (*reln*) is characterized by motor impairment (tremors and ataxia) and structural abnormalities involving the cerebellum, cerebral cortex, and hippocampus (DeSilva et al., 1997; Lossi et al., 2019). The discovery of a link between *RELN* pathogenic variants and an autosomal recessive form of lissencephaly with cerebellar hypoplasia (Lissencephaly type 2, LIS2, OMIM #257320) questioned the use of the *reeler* mouse as a model to study neuropsychiatric conditions in humans, including autism (Hong et al., 2000). Furthermore, Reelin deficiency-associated behavioral features may be difficult to measure in animal models and the data published so far mainly rely on subtle functional or structural changes (Fatemi, 2002). Therefore, the identification of a measurable biomarker for a better understanding of the pathological processes and the clinical correlations involved in *RELN*-related human conditions remains crucial (Tueting et al., 1999; Liu et al., 2001; Salinger et al., 2003; Laviola et al., 2006; Ognibene et al., 2007; Laviola et al., 2009; Lossi et al., 2019).

Additional mouse models harboring heterozygous or homozygous alterations in the *Reln* gene have been developed to investigate the potential link between Reelin and ASD (Sawahata et al., 2020). The endophenotype of *Reln* mutant mice is complex and include behavioral abnormalities that can be related to core clinical features observed in human patients (Sawahata et al., 2020). In general, rodent models of ASD have been reported to show excessive anxiety, aggressive behavior, and increased seizures susceptibility (Argyropoulos et al., 2013). More complex alterations involve sensory function and motor coordination (decreased in most models), as well as sleep and gastrointestinal functions (Argyropoulos et al., 2013). Some of these abnormalities are recapitulated in *Reln* mutant mice, supporting the association with human ASD. Increased locomotor activity in an open field test, typically reflecting increased anxiety in mouse models, was reported in Orleans hetero (BALB/C), Orleans homo (BALB/C), and ⊿C-KI (C57BL/6) mice (Lalonde et al., 2004; Sakai et al., 2016; Sobue et al., 2018; Sawahata et al., 2020). Similarly, abnormal responses in the elevated plus maze test, evaluating innate anxiety in the rodents, were described in the Orleans homo (BALB/C), and ⊿C-KI (C57BL/6) models (Lalonde et al., 2004; Sakai et al., 2016). Impaired social interaction was noticed in Orleans hetero (BALB/C), ⊿C-KI (C57BL/6), and in the recently developed Reln-del (C57BL/6) model (Sakai et al., 2016; Sobue et al., 2018; Sawahata et al., 2020). Of note, the Jackson hetero (B6C3Fe) mice exhibited instead novel object recognition deficiency, decreased fear conditioning, and reduced prepulse inhibition (Qiu et al., 2006). Abnormalities in learning and memory were further observed in the ⊿C-KI (C57BL/6) model (impaired working memory in the T-maze test) and Orleans homo (BALB/C) mice (impaired water maze test solution) (Salinger et al., 2003; Sakai et al., 2016). Impaired coordination causing abnormal responses in the stationary beams and Rotarod tests were reported in the Orleans homo (BALB/C) and in the Orleans hetero and homo (BALB/C) mice, respectively (Lalonde et al., 2004; Sobue et al., 2018). Taken together, the endophenotypes observed in *Reln* mutant mice argue in favor of the potential pathophysiological link between Reelin and ASD, helping to unravel the complex and still elusive underlying mechanisms.

The huge impact of ASD on patients’ quality of life reinforces the need for a better understanding of the underlying pathophysiological aspects. Despite several studies have been focusing on the hundreds of genes involved in brain development, either through gene-gene or gene-environment interactions, a definite understanding of the reasonably complex molecular machinery involved in autism pathogenesis is still lacking. Among the investigated genes, *RELN* certainly represents an intriguing object of study, especially considering its relevant and proteiform biological functions in human brain. The complexity of these functions likely explains the controversial results achieved by the numerous groups investigating the role of *RELN* variants in the susceptibility to ASD.

Several lines of evidence demonstrated that the number of GCCs repeats in the 5′UTR of *RELN* may play a relevant role in autism predisposition, most likely through a negative impact on protein expression and brain development. More recently, a growing evidence also emerged in favor of the association between SNPs in RELN and autism. For example, the rs736707 polymorphism (NM_173054.2:c.9606-57T > C) was detected in several heterogeneous cohorts of affected individuals from different countries, suggesting that this variant may contribute to ASD predisposition. Although this is a deep intronic variant, its possible relevance in a complex disorder such as ASD is not surprising (Wu et al., 2005; Li et al., 2008). However, the exact underlying mechanism remains elusive. It has been suggested that this SNP might affect Reelin expression through the interaction with other SNPs in *RELN* or distinct nearby loci (Li et al., 2008). However, further studies are needed to confirm this hypothesis and possibly clarify this intriguing association.

In this study, we critically reviewed *RELN* expression and haplotypes transmission in children with ASD, recapitulating the current knowledge on the contributing role of *RELN* variants in autism pathogenesis and predisposition. The conflicting evidence regarding the association Reelin-autism that emerged from our review study certainly reflects the relevant complexity of autism pathophysiology, involving variants in single genes as well as environmental factors, such as gastrointestinal abnormalities and immune imbalance (Cheroni et al., 2020). Furthermore, despite the efforts of the scientific community, the studies investigating this pathophysiological link still have undeniable limitations. For example, the behavioral abnormalities observed in animal models might be difficult to interpret and subtle ASD-related features in human subjects can be only approximated in rodent models (e.g., deficits in social interaction and communication) (Patterson, 2011). Taken together, these factors contribute to the conflicting results observed in the reported studies. However, the statistically significant link between autism and GCCs triplets in the 5′UTR or certain SNPs in *RELN* opens the route to new studies in uncharted territories (e.g., protein dosage in brain samples from affected individuals) towards an improved understanding of the pathophysiological aspects linking Reelin to ASD.
